# HbA1c-Based Score Model for Predicting Death Risk in Patients with Hepatocellular Carcinoma and Type 2 Diabetes Mellitus

**DOI:** 10.1155/2017/3819502

**Published:** 2017-01-05

**Authors:** Lingling He, Shuan Zhang, Xiaoli Liu, Yuyong Jiang, Xianbo Wang, Zhiyun Yang

**Affiliations:** Department of Traditional Chinese Medicine, Beijing Ditan Hospital and Collaborative Innovation Center of Infectious Diseases, Capital Medical University, Chaoyang, Beijing 100015, China

## Abstract

*Aim*. To establish a new score model to predict risk of death in patients with hepatocellular carcinoma and type 2 diabetes mellitus.* Methods*. This was a retrospective study of 147 patients with hepatocellular carcinoma and type 2 diabetes mellitus who came to Beijing Ditan Hospital between October 2008 and June 2013. Univariate and multivariate logistic regression analysis was performed to obtain the independent factors associated with death risk. A new score model was devised according to these factors.* Results*. A prediction score model composed of HbA1c, NLR, age, and CTP class was devised, which ranged from 0 to 7. AUROC of the score was 0.853 (*P* < 0.001, 95% CI: 0.791–0.915). Scores 0–2, 3-4, and 5–7 identified patients as low-, medium-, and high-risk categories. The cumulative survival rate was 93.6%, 83.0%, and 74.5% in the low-risk group in 1, 2, and 3 years, while it was 64.0%, 46.0%, and 26.0% in the medium-risk group, whereas it was 24.0%, 12.0%, and 6.0% in the high-risk group, respectively. The cumulative survival rate was significantly higher in the low-risk group than that in the medium-risk group and high-risk group (*P* < 0.001).* Conclusion*. The HbA1c-based score model can be used to predict death risk in patients with hepatocellular carcinoma and type 2 diabetes mellitus.

## 1. Introduction

Liver cancer is the second leading cause of cancer death worldwide in men and the most primary liver cancer is hepatocellular carcinoma (HCC) [1]. It is reported that the overall median survival time was only 6 months in patients with HCC [2]. Clinical data indicate that type 2 diabetes mellitus (T2DM) is associated with the incidence and development of hepatocellular carcinoma [3–6].

A series of score models have been established to predict risk of HCC in chronic liver diseases [7–11]. However, a score model for predicting death risk is badly in need, especially in patients with HCC and T2DM.

In this retrospective cohort study, we analyzed the baseline clinical data of patients with HCC and T2DM. We aimed to identify the independent risk factors in these patients, to establish a score model for predicting death risk, and to stratify patients for the level of care they need.

## 2. Patients and Method

### 2.1. Study Population

This was a retrospective cohort study. During the period between October 2008 and June 2013, all patients with hepatocellular carcinoma and type 2 diabetes who were first seen in Beijing Ditan Hospital, Capital Medical University, China, were followed up for the risk of death in three years. Patients who had metastatic cancer of liver, other serious concurrent illnesses, and incomplete clinical data were excluded.

### 2.2. Clinical and Laboratory Indicators

The baseline indicators were collected including a full medical history, the clinical indicators, and laboratory indexes like white blood cell (WBC), neutrophil-lymphocyte ratio (NLR), platelet (PLT), hemoglobin (HGB), liver and renal biochemistries, total cholesterol (TC), high density lipoprotein cholesterol (HDLC), glycosylated hemoglobin (HbA1c), prothrombin time (PT), and alpha fetoprotein (AFP). The transabdominal ultrasonography, computed tomography, and nuclear magnetic resonance imaging were also collected.

### 2.3. The Diagnosis of HCC

The diagnosis of HCC was mostly based on histopathological confirmation. Some patients were included by clinical diagnosis, which mean a positive lesion was detected with at least two imaging techniques (hepatic arteriography, nuclear magnetic resonance imaging, computed tomography, or abdominal ultrasonography) or detected with one imaging technique coupled with AFP level above 400 ng/ml [12].

### 2.4. Statistical Analysis

Statistical analysis was performed by SPSS version 19.0 (IBM Corp., Armonk, NY, USA). Quantitative data accorded with normal distribution were expressed in mean ± standard deviation and they were analyzed by Student's *t*-test. Data not accorded with normal distribution were expressed in median with interquartile range and they were analyzed by Mann–Whitney U test. Qualitative data were analyzed by chi-square test. Univariate and multivariate logistic regression analysis was performed to determine the variables associated with the prognosis. The risks were expressed in odd ratio (OR) and 95% confidence interval (CI). The regression coefficients were converted to integer risk scores and the final score was the sum of these values. The Kaplan-Meier method was used to estimate the cumulative survival of different groups. The log-rank test was used to compare time-to-event curves between different groups. The diagnostic value of the new score model was estimated by areas under the receiver operating characteristics (AUROC) curve. *P* < 0.05 was considered statistically significant.

## 3. Results

### 3.1. Patient Characteristics

During the study period, a total of 147 patients were included in the study. At a median follow-up time of 21 months, 96 (65.3%) of 147 patients died in three years. The 1, 2, and 3 years' cumulative survival rates were 59.9%, 46.3%, and 34.7%, respectively. The baseline characteristics of patients were shown in Table 1. Patients who died in three years were older and had higher WBC, higher NLR, lower HGB, higher total bilirubin (TBIL), higher gamma-glutamyl transpeptidase (GGT), lower serum albumin (ALB), higher creatinine (Cr), lower HDLC, higher HbA1c, longer PT, higher MELD score, and a fairly large proportion of patients with CTP class C patients (Table 1).

### 3.2. Factors Associated with Death Risk

Table 2 shows the results of univariate and multivariate logistic regression analysis. By univariate analysis, age, WBC, NLR, HGB, HbA1c, GGT, MELD score, and CTP class were found to be significant risk factors for the incidence of death. By multivariate analysis, age, NLR, HbA1c, and CTP class remained the independent risk factors associated with high risk of mortality.

**Figure 1:**
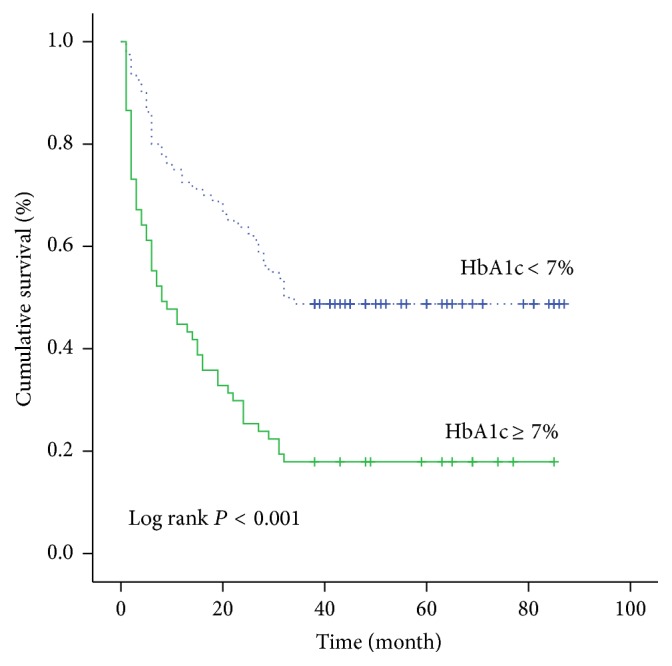
The cumulative survival rate of patients in stratified HbA1c groups.

**Figure 2:**
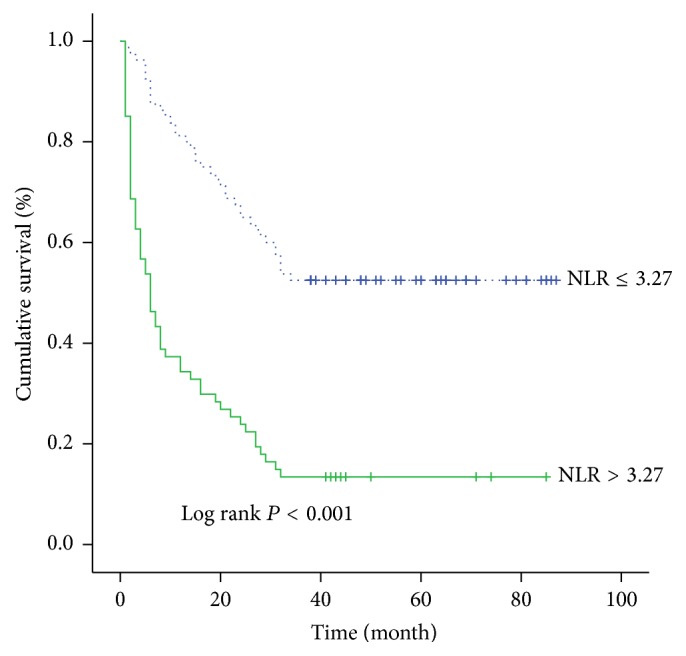
The cumulative survival rate of patients in stratified NLR groups.

**Figure 3:**
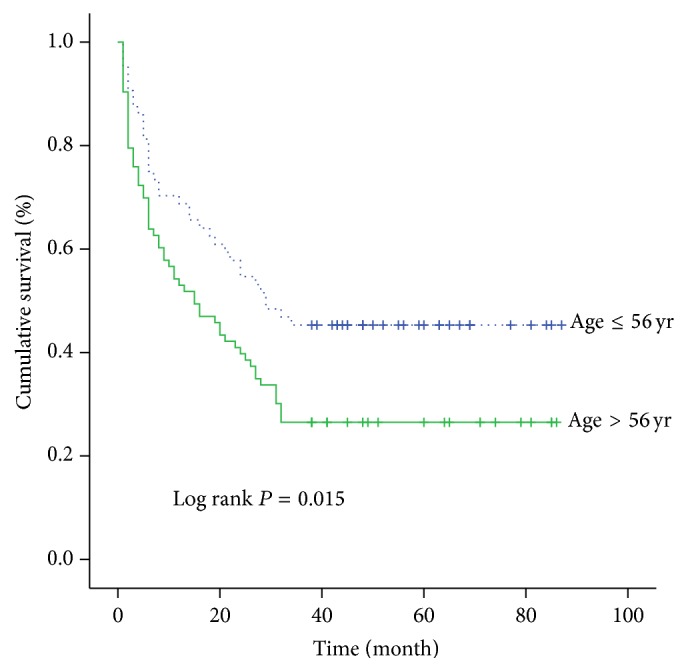
The cumulative survival rate of patients in stratified age groups.

**Figure 4:**
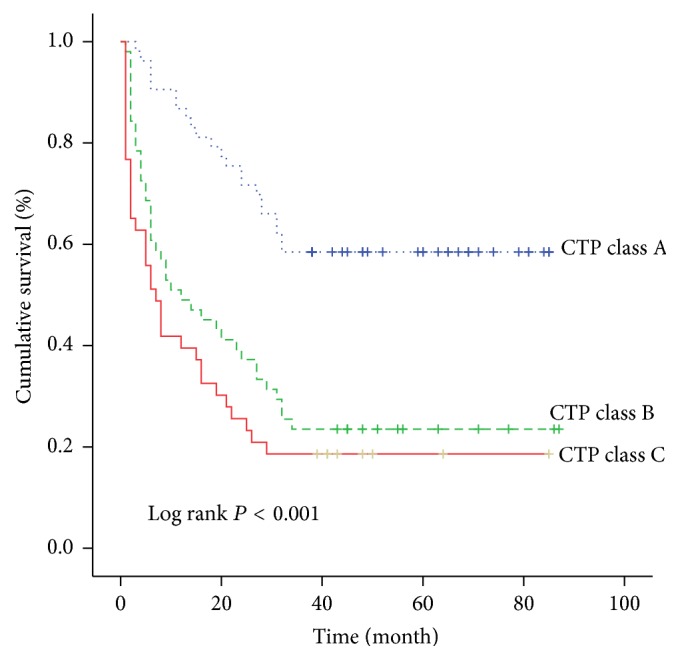
The cumulative survival rate of patients in different CTP class.

A further analysis was performed to evaluate the influence of each risk factor on the cumulative survival rate. The 3-year cumulative survival rate was 48.8% in HbA1c < 7% group while it was 17.9% in HbA1c ≥ 7% group (Figure 1). It was significantly higher in HbA1c < 7% group than that in HbA1c ≥ 7% group. Similarly, the 3-year cumulative survival rate was 52.5% in the NLR ≤ 3.27 group while it was 13.4% in the NLR > 3.27 group (Figure 2). The 3-year cumulative survival rate was 45.3% in the age ≤ 56 year group while it was 26.5% in the age > 56 year group (Figure 3). The 3-year cumulative survival rate was 58.5% in CTP class A group, whereas it was 23.5% in CTP class B group and 18.6% in CTP class C group (Figure 4). It was significantly higher in CTP class A group than that in CTP class B group and CTP class C group.

### 3.3. Prediction Score Model

**Figure 5:**
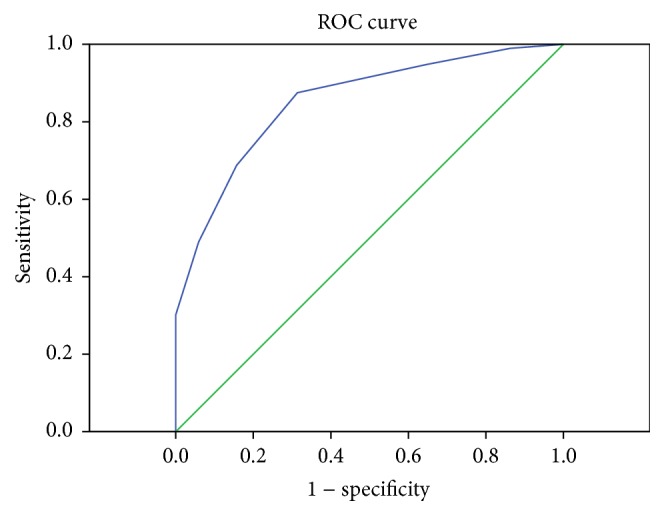
AUROC of HbA1c-based score model.

**Figure 6:**
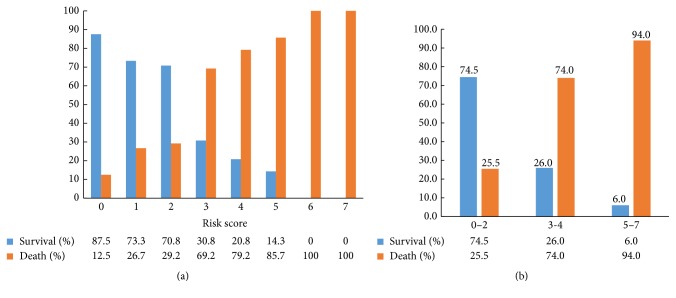
(a) The survival and mortality rate of patients in each score. (b) The survival and mortality rate of patients in stratified score groups.

A new score model was devised using independent risk factors in the multivariate logistic regression analysis. It was attributed to each parameter according to its relative contribution, as determined by the regression coefficients (Table 3). The score ranged from 0 to 7. AUROC was 0.853 (*P* < 0.001, 95% CI: 0.791–0.915) (Figure 5). We observed that the survival rate gradually decreased with increasing score, from 87.5% in score 0, 73.3% in score 1, 70.8% in score 2, 30.8% in score 3, 20.8% in score 4, and 14.3% in score 5 to 0.0% in scores 6 and 7 (Figure 6(a)).

We assigned the scores 0–2, 3-4, and 5–7 to low-, medium-, and high-risk categories; thus, 47, 50, and 50 patients belonged to each category, respectively. In the low-risk group, 35 (74.5%) patients survived in three years after they were diagnosed with HCC and T2DM. Correspondingly, the patients who survived in the medium-risk group and high-risk group were 13 (26.0%) and 3 (6.0%), respectively (Figure 6(b)).

**Figure 7:**
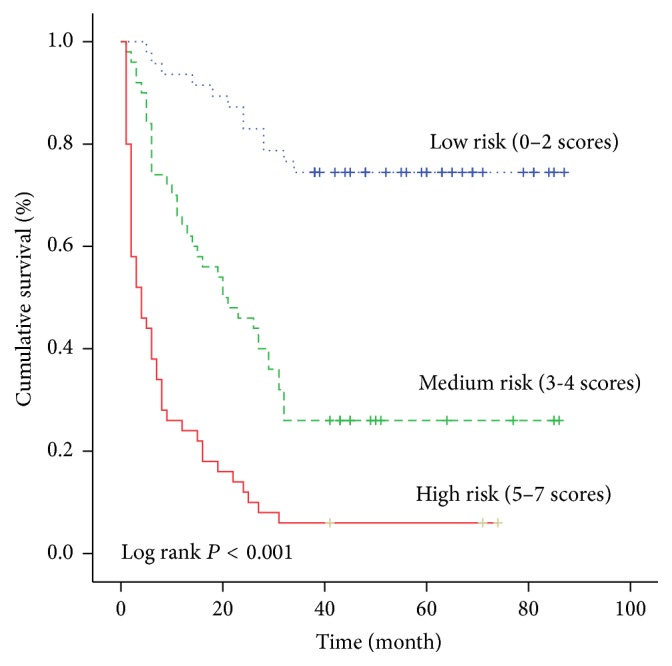
The cumulative survival rate of patients in different risk groups.

The cumulative survival rate was 93.6%, 83.0%, and 74.5% in the low-risk group in 1, 2, and 3 years, while it was 64.0%, 46.0%, and 26.0% in the medium-risk group, whereas it was 24.0%, 12.0%, and 6.0% in the high-risk group, respectively. The cumulative survival rate was significantly higher in the low-risk group than that in the medium-risk group and high-risk group (*P* < 0.001) (Figure 7). We also observed that the death risk in low-, medium-, and high-risk categories was increased significantly because the odds ratio of these catigories increased multiply (Table 4).

## 4. Discussion

In this study, we established the risk score model based on HbA1c to predict death risk in patients with hepatocellular carcinoma and type 2 diabetes mellitus. This model was not only accurate and sensitive to predict 3-year mortality, but also simple and easy to be applied in the clinical practice as it was composed of four routinely available clinical and laboratory parameters. Patients in different risk group had distinctly different risk of death. The 3-year cumulative survival rate was 74.5% in the low-risk group while it was just 6.0% in the high-risk group. Predicting the risk of death accurately could let clinicians pay more attention to the high-risk group and give them more appropriate medical care. With this method, the health care resources could be allocated and utilized more efficiently.

There have been various kinds of risk models to predict HCC in chronic liver diseases [7–11]. In several models, diabetes is one of the variables [10, 11]. It is also reported that history of diabetes is associated with deaths from HCC [13]. Patients with DM showed significantly lower overall survival than those without DM [14, 15]. Thus, a specific group of people with HCC and T2DM should be observed. To our knowledge, this is the first study to establish a prediction score model for death risk in the specific population of HCC and T2DM.

HbA1c is a biomarker of the blood glucose level over the last 3 months and it is believed to be the robust indicator of diabetes management. Recent studies have shown that increased serum HbA1c level is associated with the development and progression of cancer [16]. Furthermore, higher HbA1c level is correlated with higher risk of all-cause mortality in cancer patients [17–20]. In pancreatic cancer patients with diabetes, survival time of HbA1c ≥ 7.0% group was significantly higher than that of HbA1c < 7.0% group [21]. The mortality risk was twice as high in breast cancer patients with HbA1c ≥ 7.0% compared with HbA1c < 6.5% patients [22]. In our study, HbA1c is the most important parameter in the risk score model. The median survival time in HbA1c ≥ 7.0% group was 33 months compared with 8 months in HbA1c < 7.0% group. The risk of mortality was eight times higher in HbA1c ≥ 7.0% group than that in HbA1c < 7.0% group (OR: 8.976, 95% CI: 3.164–25.460). These results add to evidence that any clinical intervention should be put into practice to reduce serum HbA1c level to reduce mortality risk.

NLR has recently been reported to predict prognosis of HCC in several studies [23, 24]. Higher NLR level was associated with poor disease-free survival and overall survival [25–27]. Our study found that NLR > 3.27 may be an indicator of poor overall survival in patients with HCC and T2DM. The median survival time in NLR > 3.27 group was 38 months, while it was only 6 months in NLR ≤ 3.27 group.

Age is believed to be an independent factor associated with prognosis in most studies and it is an important parameter in a variety of score models [7–11]. In the present study, patients under age of 56 have longer median survival time than patients older than 56 (29 versus 15 months).

Numerous studies have compared the power of Child-Turcotte-Pugh class with MELD score in predicting prognosis in patients with liver disease [28–30]. The current study confirmed that CTP class was one of the independent risk factors in the multivariate analysis in patients with HCC and T2DM, whereas MELD score was not. Nevertheless, there is no significant difference between CTP class B group and CTP class C group in terms of 3-year cumulative survival rate (*P* = 0.188). Furthermore, the risk of mortality in CTP class B and C groups were nearly the same when compared to CTP class A group (OR: 5.662, 95% CI: 1.961–16.343 and OR: 5.567, 95% CI: 1.711–18.118).

The potential limitations of our study should be clarified. One is that the variables analyzed in this study were baseline covariates and the treatment strategy was not taken into account. More still needs to be done to optimize this score model that may trigger different clinical interventions. The other is that the population included in this study is small that a further validation of a large prospective cohort study is necessary to evaluate the prediction accuracy.

In conclusion, HbA1c, NLR, age, and CTP class are independently correlated with death risk of patients with HCC and T2DM. A novel score model based on HbA1c has been formulated and it is of great use to permit clinicians to identify the individual risk of death and to stratify these patients for the level of care they need.

## Figures and Tables

**Table 1:** Characteristics of patients.

Variable	Total patients (*N* = 147)	Survival group (*N* = 51)	Death group *N* = 96	*P* value
Age (yr)	58.93 ± 9.50	56.80 ± 10.15	60.06 ± 8.98	0.050
Male sex	116 (78.9)	42 (82.4)	74 (77.1)	0.456
History of smoking	57 (40.1)	23 (45.1)	34 (35.4)	0.288
History of alcohol use	47 (32.0)	16 (31.4)	31 (32.3)	1.000
Family history of liver disease	35 (23.8)	11 (21.6)	24 (25.0)	0.689
Family history of diabetes	6 (4.1)	3 (5.9)	3 (3.1)	0.418
WBC (×10^9^/L)	5.0 ± 2.7	4.4 ± 2.1	5.3 ± 2.9	<0.001
NLR	3.0 (1.8, 5.3)	1.9 (1.5, 3.1)	4.1 (2.1, 6.7)	<0.001
PLT (×10^9^/L)	79.8 (53.4, 148.9)	83.0 (59.0, 122.3)	79.2 (50.8, 156.8)	0.880
HGB (g/l)	116.1 ± 23.5	123.9 ± 24.0	112.0 ± 22.3	<0.001
ALT (U/L)	33.7 (22.5, 55.1)	32.6 (21.5, 50.5)	34.3 (25.2, 59.4)	0.490
TBIL (*μ*mol/l)	21.9 (12.3, 42.1)	15.8 (9.7, 26.3)	29.6 (15.2, 64.6)	<0.001
GGT (U/L)	70.5 (34.0, 146.2)	49.5 (29.0, 96.3)	86.5 (42.3, 198.5)	<0.001
ALB (g/l)	33.7 ± 6.3	37.2 ± 6.0	31.9 ± 5.6	<0.001
TC (mmol/L)	3.5 (2.8, 4.2)	3.8 (3.0, 4.2)	3.3 (2.8, 4.1)	0.170
HDLC (mmol/L)	1.0 ± 0.5	1.2 ± 0.4	0.9 ± 0.5	<0.001
HbA1c (%)	7.0 ± 2.0	6.4 ± 1.9	7.4 ± 2.0	0.010
Cr (*μ*mol/l)	64.0 (54.0, 77.0)	62.0 (53.0, 71.0)	66.0 (54.3, 85.3)	0.040
PT (s)	14.1 ± 2.8	13.4 ± 2.7	14.5 ± 2.8	<0.001
AFP (ng/ml)	15.2 (4.7, 169.2)	16.8 (4.6, 83.3)	15.1 (5.5, 280.8)	0.390
MELD score	22.4 ± 7.0	19.8 ± 5.6	23.8 ± 7.3	<0.001
CTP class (A/B/C)	53/51/43	31/12/8	22/39/35	<0.001

**Table 2:** Factors associated with death risk in patients with HCC and T2DM.

Variable	Univariate			Multivariable		
	*P* value	Odds ratio	95% CI	*P* value	Odds ratio	95% CI
Age	0.050	1.038	1.000–1.078	0.043	1.048	1.001–1.096
WBC	0.049	1.161	1.001–1.346			
NLR	<0.001	1.398	1.168–1.673	0.015	1.258	1.046–1.514
HGB	0.005	0.977	0.962–0.993			
HbA1c	0.006	1.315	1.080–1.602	0.005	1.362	1.096–1.693
GGT	0.011	1.005	1.001–1.009			
MELD score	0.001	1.101	1.038–1.168			
CTP class	<0.001	2.714	1.668–4.414	0.006	1.312	1.083–1.590

**Table 3:** Components of the score model.

Variable	Regression coefficient	*P* value	Odds ratio	Risk score
Age ≥ 57 (yr)	1.317	0.005	3.734	1
HbA1c ≥ 7 (%)	2.195	<0.001	8.976	2
NLR ≥ 3.27	1.905	<0.001	6.72	2
CTP class A				
CTP class B	1.734	0.001	5.662	2
CTP class C	1.717	0.004	5.567	2

**Table 4:** Death risk in stratified score groups.

Score	Regression coefficient	*P* value	Odds ratio	95% CI
0–2			Reference	
3-4	2.116	<0.001	8.301	3.339–20.635
5–7	3.822	<0.001	45.694	11.981–174.281
